# What Is the Correlation between Preeclampsia and Cancer? The Important Role of Tachykinins and Transition Metal Ions

**DOI:** 10.3390/ph16030366

**Published:** 2023-02-28

**Authors:** Klaudia Szczerba, Kamila Stokowa-Soltys

**Affiliations:** Faculty of Chemistry, University of Wroclaw, F. Joliot-Curie 14, 50-383 Wroclaw, Poland

**Keywords:** cancer, preeclampsia, tachykinins, transition metal ions, metal complexes, oxidative stress, angiogenesis

## Abstract

Metal ions are irreplaceable in many biological processes. They are components of numerous metalloproteins and serve as cofactors or structural elements for enzymes. Interestingly, iron, copper and zinc play important roles in accelerating or preventing neoplastic cell transformation. Noteworthily, a lot of proliferative and invasive mechanisms are exploited by both malignant tumors and pregnancy. Cancer cells, as well as developing placenta cells, create a microenvironment supportive of immunologic privilege and angiogenesis. Therefore, pregnancy and cancer progression share many similarities. Moreover, during preeclampsia and cancer, significant changes in relevant trace element concentrations, tachykinin levels, expressions of neurokinin receptors, oxidative stress and angiogenic imbalance are observed. This sheds a new light on the role of metal ions and tachykinins in cancer progression and pregnancy, especially in preeclamptic women.

## 1. Introduction

Metal ions are inorganic components considered to be essential nutrients for all kingdoms of life [1], because they are irreplaceable in many biological processes [2]. These essential elements are divided into two groups on the basis of their requirement, namely macroelements (such as Ca, Mg, etc.) and microelements (such as Cu, Zn, Fe, etc.) [3]. Microelements, also known as trace elements, constitute less than 0.01% of human body mass. This group includes chromium, cobalt, copper, fluorine, iodine, iron, manganese, molybdenum, selenium and zinc, which are absorbed from the gastro-intestinal tract and stored in the liver [4]. Most importantly, metal ions are involved in diverse biochemical reactions by acting as structural components of enzymes or cofactors, and control biological processes by binding to the receptors and transcription factors [5]. Trace elements play important roles in the regulation of gene expression, immune functions and antioxidant defense. Thus, maintaining their homeostasis in the tissues is crucial for normal physiological function and development [4]. Excess, as well as deficiency, and the abnormal distribution of metal ions will seriously affect various physiological properties of cells, tissues and organs [6,7]. Metal ions, which are necessary to the proper functioning of the human body, may also induce many diseases, including cancer. Too high concentrations of some trace elements lead to various harmful intracellular events such as oxidative stress, DNA fragmentation and mitochondrial dysfunction, which are associated i.a. with tumorigenesis [8,9,10,11]. Especially important trace elements for the growth and proliferation of cancer cells are iron, copper and zinc, due to their role in preventing or accelerating neoplastic cell transformation and in modulating the pro-tumorigenic and inflammatory response in immune cells, such as macrophages [5,7].

Beyond any doubt, iron is an indispensable metal, the most abundant trace element in the human body [12]. However, there is no free iron inside the cell. Only a small fraction of loosely bound iron can be found in the cytoplasm [13]. In living matter, iron exists in two stable oxidative states: Fe(II), called ferrous, and Fe(III)—ferric. In aqua solution, Fe(II) is spontaneously oxidized to Fe(III) by molecular oxygen, which results in the precipitation of Fe(OH)_3_ [14]. Under pathological conditions, iron homeostasis may be disrupted at different levels. This metal serves as a cofactor for numerous hemoproteins and non-heme iron-containing proteins, including many enzymes. Therefore, iron is involved in oxygen binding and transport (hemoglobin), oxygen metabolism (catalases, peroxidases), cellular respiration and electron transport (cytochromes) [15,16]. Cancer cells display dysregulated iron homeostasis and they require more iron for their metabolism and growth. Iron is necessary in the early stages of tumor development, the proliferation of neoplastic cells and the promotion of metastasis [17,18]. Due to the overexpression of many iron-related genes, malignant tumors compete with the liver and spleen for iron storage. This leads to inadequate erythropoiesis and anemia in 40–70% of cancer patients [17,19]. Iron uptake in cancer cells is increased through the upregulation of Transferrin/Transferrin-receptor (Tf/TfR), and Lipocalin-2/Lipocalin-2 Receptor (Lcn-2/Lcn-2R) systems and also Divalent Metal Transporter-1 (DMT1) [20,21]. Elevated iron pools in cytosol provide to CyclinD1/CDK4 overexpression p21 down regulation and perturbations in the global histone and DNA methylation, which supports cellular proliferation and survival functions of cancer [22,23]. Simultaneously, mitochondrial uptake is increased via Mitoferrin-2 (Mfrn-2) and upregulated Frataxin [24,25]. Since iron overloading can cause lipid peroxidation, cancer cells reduce the risk of this process by Selenoprotein Glutathione Dependent Peroxidase 4 (GPX4) activity. GPX4 protects the mitochondrial metabolism from Reactive Oxygen Species (ROS)-induced membrane damage due to reducing the amount of intracellular radicals [26,27]. It is worthy of interest that tumor-associated macrophages (TAMs), which are involved in the maintenance of a pro-tumorigenic microenvironment and in facilitating metastatic dissemination, also adopt a strong iron-release phenotype that contributes to tumor cell proliferation and growth [21,28].

Although intracellular copper concentration is around 10-fold lower than that of iron [29], this transition metal ion is a necessary catalytic cofactor of many enzymes such as Cytochrome C Oxidase (Cyt C Oxidase), Superoxide Dismutase 1 and 3 (SOD1 and SOD3), Glutathione Peroxidase (GPx) and Ceruloplasmin. Because copper exists in two oxidation states, cuprous (Cu(I)) and cupric (Cu(II)), this metal ion is involved in the mitochondrial electron transport chain, the detoxification of ROS, the conversion of hydroperoxides into hydroxides and in ferroxidation, respectively [30]. Cupric ions are some of the most stable divalent transition metal ions and possess the highest affinity for metalloproteins according to the Irving–Williams stability series [31]. Substantially, this metal also binds to proteins directly involved in cancer progression. Since copper is essential for Cyt C Oxidase, this micronutrient must be delivered to the mitochondria. This process relies on the specialized transport protein called Phosphate Carrier SLC25A3 and on the copper pool in the endoplasmic reticulum. Interestingly, SLC25A3 overexpression promotes Cytochrome C release and apoptosis and has been associated with chronic myeloid leukemia progression [32,33]. Furthermore, in human cells, copper binds to Dual Specificity Mitogen-Activated Protein Kinase Kinase 1 (MEK1), which is involved in the RAS-RAF-MEK-ERK pathway, required for cell proliferation and tumorigenesis [34]. Moreover, this trace element also modulates cancer-related proteins expression or activation indirectly. Copper inhibits prolyl hydroxylase, therefore stabilizing Hypoxia-inducible factor 1 (HIF-1) and increasing the transcription of genes involved in angiogenesis and the epithelial to mesenchymal transition [30,35,36]. This metal ion also increases the production of the vasodilator nitric oxide (NO) by activating the endothelial Nitric Oxide Synthetase (eNOS) [37]. Moreover, the copper-dependent enzyme lipoxygenase (LOX) catalyzes the cross-linking of collagen and elastin in the extracellular matrix (ECM) and interacts with the Mediator of Cell Motility 1 (MEMO1), which is involved in cancer cell migration [38].

After iron, zinc is the second most abundant transition metal ion in all living organisms. It plays a structural, regulatory and catalytic role in numerous proteins. In contrast to other transition metal ions, it does not participate in redox reactions [39]. Zinc is a nutritionally fundamental trace element which is required for the activity of over 300 enzymes (e.g., carbonic anhydrases, carboxypeptidase, matrix metalloproteinases), acts as a cofactor for some antioxidant enzymes (SOD1, SOD3), and is essential for the activity of transcription factors (interferon-regulatory factors, Nuclear Factor-κB) and proteins regulating gene transcription (S100 protein family) [40,41,42]. Zinc is considered to be critical in the host defense against the initiation and progression of carcinogenesis. Moreover, the intake of dietary zinc (at plasma concentrations not exceeding 30 μM) is associated with a reduced risk of breast, colorectal, esophageal, gastric and prostatic cancer [43,44]. There are two families of Zn transporters which contribute to cancer in opposite ways: ZnTs (SLC30) and ZIPs (SLC39). ZnTs reduce cytoplasmic zinc concentrations, whereas ZIPs enhance the level of zinc ions [42]. Interestingly, in some cancer types (e.g., highly aggressive and metastatic basal breast cancer, esophageal cancer), the overexpression of zinc transporters ZnTs was observed. Contrarily, in some other cases (advanced prostatic cancer, pancreatic cancer), ZnTs expression was decreased [43,45]. However, in most cancers, the expression of ZIPs transporters is elevated. The over-expression of ZIP1, ZIP6 and ZIP7 is associated with Zn hyper-accumulation in breast tumors and several breast cancer cell lines. Elevated ZIP6 is responsible for lower aggressiveness of tumors [45]. However, ZIP1-3s are downregulated in prostate cancer tissues, which leads to low zinc levels in the prostate gland [46]. Accordingly, the activity of the Mitochondrial Aconitase (ACO2) and Cytochrome C Reductase (Cyt C Reductase) increases, with consequent intensified citrate oxidation and respiration, as well as a high rate of proliferation and invasiveness [47]. Inversely, in pancreatic cancer tissues, all ZIP proteins with the exception of ZIP4 are downregulated. Notwithstanding, it also leads to lower intracellular Zn concentrations, and thus provides cancer cells with increased resistance to the cytotoxic effects of Zn [48,49]. Moreover, zinc might affect the signaling between immune cells and cancer cells by altering the membrane structure and receptor expressions; however, our understanding of the significance of the role of zinc homeostasis in the regulation of the immune system and tumors is still meager [5,44].

Many proliferative, invasive, and immune tolerance mechanisms are exploited by both malignant tumors and pregnancy. Beyond any doubt, pregnancy and the associated changes are a normal physiological process in response to the development of the fetus. However, both cancer cells and developing placenta cells create a microenvironment supportive of immunologic privilege and angiogenesis. The main reason is to establish a nutrient supply and edit or avoid the host immune response [50]. Obviously, as the pregnancy progresses, metabolic demand increases due to the requirements of the growing fetus [51]. Maternal nutritional status is directly associated with fetal growth and development; hence, trace elements deficiency can lead to adverse pregnancy outcomes [52]. Nevertheless, overexposure to certain trace elements could be detrimental to the health of both pregnant women and child in the womb [53]. One of the disorders affecting pregnant women is preeclampsia (PE). PE is a multifactorial blood pressure disorder characterized by high maternal blood pressure and proteinuria. The causes of that disorder have not yet been fully discovered [54,55]. Noteworthily, in PE a significantly elevated copper pool in blood plasma and increased levels of serum iron are observed. Notwithstanding, the zinc concentration in blood plasma remains comparable to that of healthy women; however, the Cu/Zn ratio increases significantly [56,57,58]. PE also carries a certain risk of cancer. Recent studies lead to the conclusions that women with preeclampsia have a lower breast cancer risk regardless of their menopausal status, but at the same time a higher ovarian cancer risk compared with the woman with normal pregnancy [59]. Furthermore, during PE the balance between angiogenic and anti-angiogenic factors is disturbed. A noticeably higher level of Soluble Fms-like Tyrosine Kinase-1 (sFlt-1), a decoy receptor for Vascular Endothelial Growth Factor (VEGF), which may bind to Placental Growth Factor (PlGF) and VEGF and block their angiogenic function, is observed [55,60]. Interestingly, significantly elevated Neurokinin B (NKB) levels in peripheral blood and umbilical cord blood were also found in preeclamptic women [61]. NKB plays a key role in the proper functioning of the female organism during pregnancy due to its essential role for the regulation of the blood flow to the placenta [62,63]. NKB belongs to the tachykinin neuropeptide family, which also includes i.a. Neurokinin A (NKA) and Substance P (SP). They interact with tachykinin receptors NK1R, NK2R and NK3R [64,65]. In PE, the tachykinin gene-related peptides were identified as secreted factors from the placenta in excessive amounts [66]. Tachykinins and their receptors are also important in the process of carcinogenesis. NKB has an anti-angiogenic effect by initiating multiple actions that jointly prevent vascular remodeling [37]. Moreover, analogues of NKB demonstrate strong antitumor activities with no significant side effects in vivo [67]. Further, high NK2R gene expression is correlated with the poor survival of colorectal cancer patients, and NK2R antagonist administration is responsible for the inhibition of tumorigenesis [68]. Additionally, the polymorphism of NK2R predicts lymph node metastasis in colorectal cancer patients [69]. NK1R is overexpressed in tumors and, due to SP binding, activates tumor cell proliferation, angiogenesis and tumor cell migration for invasion and metastasis. Therefore, NK1R antagonists specifically inhibit all these processes [70]. Moreover, NK1R promotes non-small cell lung cancer progression through the transactivation of Epidermal Growth Factor Receptor (EGFR) [71] and acts as a promising biomarker and therapeutic target in breast cancer [72].

During PE and cancer diseases, changes in relevant trace element concentrations, oxidative stress and angiogenic imbalance are observed [9,55,56,73,74,75,76,77,78]. Further, preeclamptic women have higher ovarian cancer risk compared to women during normal pregnancy [61]. Additionally, tachykinins play an interesting role in these two diseases. The similarities and correlations between cancer and preeclampsia led us to dive deeper into the topic. The review reveals interactions between tachykinins and metal ions, as well as their importance in cancer progression and preeclampsia.

## 2. Overall Characteristics of Cancer

**Figure 1:**
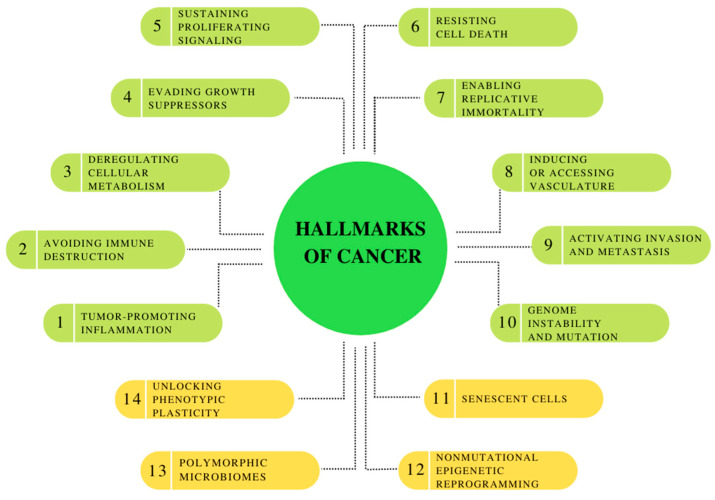
Hallmarks of Cancer: the canonical hallmark capabilities and enabling characteristics, with prospective new additions: unlocking phenotypic plasticity, nonmutational epigenetic reprogramming, polymorphic microbiomes and senescent cells. Own elaboration based on [82].

Cancer is one of the leading causes of death worldwide. According to the International Agency for Research on Cancer, 19.3 million new cases and 10.0 million cancer-related deaths were reported in 2020 [79]. By 2040, the number of new cancer cases per year is expected to grow to 28.4 million [80]. A major challenge in specific diagnosis and treatment effectiveness is that cancers are heterogeneous diseases at histological, biological, pathological, and molecular levels [81]. However, there are some common features of all cancers. The latest hallmarks of cancer (Figure 1) embody eight hallmark capabilities (i.a., inducing/accessing vasculature, activating invasion and metastasis and deregulating cellular metabolism) and two enabling characteristics (tumor-promoting inflammation and genome instability and mutation), which are fundamentally involved in activating hallmark functional capabilities necessary for tumor growth and progression. Moreover, this collection currently incorporates four additional proposed emerging hallmarks and enabling characteristics: senescent cells, nonmutational epigenetic reprogramming, polymorphic microbiomes and unlocking phenotypic plasticity [82].

**Figure 2:**
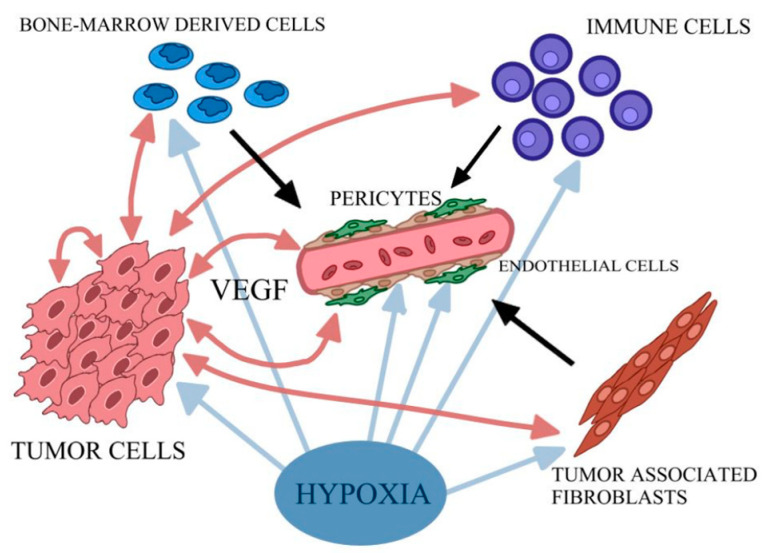
Simplified scheme of cascade of events triggered by hypoxia. Tumor cells produce VEGF and other pro-angiogenic factors, and a variety of pro-inflammatory cytokines stimulating endothelial cells to proliferate. Tumor-associated fibroblasts and bone-marrow-derived angiogenic cells additionally stimulate the endothelial cells. The creation of stable vessel interplay with pericytes is also needed. Own elaboration based on [85].

Moreover, the development of neoplastic disease is a multistep process basically involving mutation and the subsequent selective expansion of the mutated cell. At all stages of carcinogenesis, oxidative stress (OS) and oxidative damage occur due to the overproduction of ROS, which include a group of molecules derived from molecular oxygen, such as hydrogen peroxide (H_2_O_2_), superoxide anion radical (O_2_^•–^), and the most reactive among other ROS members: hydroxyl radical (^•^OH) [9,83]. They influence cancer evolution, either initiating/stimulating tumorigenesis and supporting the transformation/proliferation of cancer cells or causing cell death. Furthermore, oxidative DNA damage is a major cause of mutations [9,84]. Moreover, angiogenesis is mandatory to sustain the deregulated proliferation of tumor cells. The rapid expansion of the tumor mass causes hypoxia—a lack of oxygen inside the tumor. Therefore, in response to hypoxia, the angiogenic program is switched on (Figure 2). Together with the lack of nutrients, it bolsters the expression of inflammatory signals and cytokines that recruit vascular cells for the tumor vessel plexus formation [78,85].

**Figure 3:**
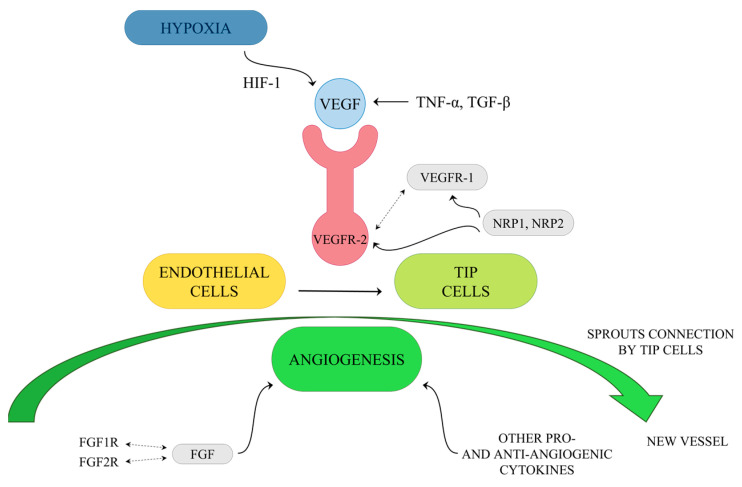
Simplified diagram of tip cells activation by VEGF.

Angiogenesis mainly involves the breakdown of the vascular extracellular matrix for subsequent endothelial cell invasion. One of the key mediators of the tumor blood vessels forming is VEGF. Hypoxia-induced transcription of VEGF mRNA is mediated by the binding of HIF-1 and modulated by cytokines. VEGF production can also be enhanced by Tumor Necrosis Factor α (TNF-α) and Transforming Growth Factor β (TGF-β). VEGF may interact with three tyrosine kinase receptors: VEGF receptor-1 (VEGFR-1), VEGFR-2 and VEGFR-3. The first two of them are predominantly expressed on vascular endothelial cells. Ligands of VEGFR-1 include VEGF-A, -B and PIGF, while those of VEGFR-2 include VEGF-A, -C and -D [85,86]. Upon activation of VEGFR-1 and VEGFR-2, which cooperate directly through heterodimerization, changes in endothelial cell plasticity in the angiogenic sprout are initiated and followed by a vascular signal. As a result of this process, the activation of endothelial cells as tip cells occurs (Figure 3). Different sprouts connect with each other by tip cells to form lumen and form new vessels, and then endothelial cells undergo quiescence followed by stabilization and maturation by mural cells [85,87]. The described formation of blood vessels in the tumor ecosystem occurs through Sprouting Angiogenesis, which stands for the formation of new vascular structures from a preexisting vessel network. Less frequently, mechanisms for neovascularization include vasculogenesis (the de novo formation of blood vessels as a consequence of vascular progenitor cell differentiation), vasculogenic mimicry, and intussusception [78]. Ultimately, tumor-associated vessels contribute to the dissemination of tumor cells by abetting their entry into the circulatory system and aiding in the generation of the pre-metastatic niche [78]. Interestingly, VEGF receptors have co-receptors such as Neuropilin-1 (NRP1) and Neuropilin-2 (NRP2) [88,89]. They are presented on tumor-associated vessels and expressed by a variety of cancers such as lung or brain tumors [90,91]. Additionally, members of the Fibroblast Growth Factors (FGF) family are known to be angiogenic activators. They interact with its receptors, FGF1R and FGF2R, and presumably participate in the development of resistance to VEGF targeting agents [92]. Thus, searching for novel therapeutics and targets for anti-angiogenesis therapy is crucial, especially in light of disappointing results of clinical applications of strategies acting on the VEGF axis, such as tumors evolving mechanisms of resistance or further severe side effects of anti-angiogenic drugs, such as bleeding and chemoresistance. Furthermore, the key to inhibit cancer progression lies in combining therapies targeting stromal components with traditional anticancer medication [93,94,95,96].

## 3. Clinical Characterization of Preeclampsia

Preeclampsia is a multifactorial blood pressure disorder, a quite common complication of pregnancy. This disease encompasses 8% to 10% of pregnant women and each year leads to over 50,000 deaths worldwide [55,97]. The main symptom of this condition is sustained high maternal blood pressure (high systolic/diastolic blood pressure of ≥140/90 mm Hg), often accompanied by proteinuria (≥300 mg/24 h after 20 weeks of gestation in women with previously normal blood pressure) [54,55]. PE affects both mother and fetus. On the one hand, it leads to maternal organ dysfunction, e.g., acute renal insufficiency, liver, neurological or hematological complications. It causes renal or liver failure, Hemolysis, Elevated Liver enzymes and Low Platelets (HELLP) syndrome, and cerebral oedema with seizures. On the other hand, in the fetus, PE induces, i.a., iatrogenic prematurity, fetal growth restriction, oligohydramnios, and stillbirth. The cause of neonatal or fetal death is placental abruption, preterm delivery or intrauterine death [98]. 

There are two types of preeclampsia: maternal (late-onset) and, more severe, placental (early-onset) preeclampsia [99]. Maternal PE is based on the interaction between a healthy placenta and maternal factors due to maternal endothelial dysfunction. This results in microvascular damage [55,100]. Placental PE is related to abnormal placentation due to placental hypoxia. It is associated with the alteration in the physiologic transformation of spiral arteries in cytotrophoblast, thus causing blood flow resistance in the uterine arteries. Placental hypoxia leads to decreased PlGF and disturbs the balance between angiogenic and anti-angiogenic factors. Attention was drawn to an elevated sFlt-1 level, a decoy receptor for VEGF, which may bind to PlGF and VEGF and block their angiogenic function [55,60]. Moreover, sFlt-1/PlGF ratio was considered as a high predictive value for preeclampsia development [101,102,103,104]. Placental hypoperfusion and ischemia are associated with a high level of OS. During pregnancy, the placenta undergoes a state of OS due to increased mitochondrial activity in the placenta that leads to the overproduction of ROS. This phenomenon is much more severe in PE than during normal pregnancy [73,105]. Women suffering from PE have higher levels of lipid peroxides and lower levels of essential antioxidants (e.g., vitamins C, A, E and glutathione) in a comparison group of healthy pregnant women [75,106]. Furthermore, the iron-binding capacity is also lowered in preeclamptic women. Moreover, it is suggested that the Angiotensin AT1 Receptor (AT1-AA), through the activation of Nicotinamide Adenine Dinucleotide Phosphate (NADPH) Oxidase, could contribute to ROS production and inflammatory responses in preeclampsia [107]. Placental hypoxia is also considered to be triggering factor for reduced NO synthesis, which is a potent vasodilator that helps in the relaxation of spiral arteries, therefore reducing vascular resistance [108].

## 4. Preeclampsia in Terms of Neoplasia

The association of preeclampsia with the risk of cancer was investigated by many cohort studies [59,109,110]. It was controversial to define the unequivocal influence of a history of preeclampsia on the cancer incidence rate. On the one hand, research suggested a higher risk of all types of cancer in women developing preeclampsia [110]. On the other hand, scientists concluded that preeclampsia is associated with a decreased risk of premenopausal and HER2-enriched breast cancer [111]. However, the latest reports suggest that the difference in the total cancer risk between the control and preeclampsia groups was statistically nonsignificant; however, the women with preeclampsia have a lower breast cancer risk regardless of their menopausal status and higher ovarian cancer risk compared with the woman with normal pregnancy [59]. 

During pregnancy, the physiological level of NKB in the blood increases to 0.08 nM [112]. Interestingly, significantly elevated NKB levels, 0.53–0.92 nM in peripheral blood and 1.42–2.35 nM in umbilical cord blood, were found in preeclamptic women [61]. NKB belongs to the tachykinin neuropeptide family, which includes, i.e., NKA and SP. These neuropeptides interact with G protein-coupled mammalian tachykinin receptors: NK1R, NK2R and NK3R. Their potency of affinity changes as follows: NK1R–SP > NKA > NKB, NK2R–NKA > NKB > SP and NK3R–NKB > NKA > SP [64,65]. Tachykinin receptors are expressed in the tumor microenvironment in many cancer types and have a significant impact on the biochemistry of tumor growth [113,114,115,116,117]. NKB plays a key role in the proper functioning of the female reproductive system, especially during pregnancy, and is essential for the regulation of the blood flow to the placenta [62,63]. Interestingly, the investigation of the NKB anti-angiogenic effect has shown a conservative mechanism in which NKB initiates multiple activities that jointly resist vascular remodeling. By hydrolysis of phosphoinositide, tachykinins lead to the production of metabolites that mobilize intracellular Ca^2+^, which plays an important role in the regulation of cell motility. As NKB mediates the ablation of Ca^2+^ oscillations, and the elevation of 3′–5′ cyclic adenosine monophosphate (cAMP) reduces VEGFR-1 and VEGFR-2 expression, cellular proliferation and migration, and induces the antiangiogenic protein, calreticulin. Noteworthily, NKB activity is significantly enhanced by thromboxane A2 (TXA2) signaling, while TXA2 is also overexpressed in women with PE. Furthermore, the disruption of NKB signaling stimulates angiogenesis comparable to FGF2 [118]. In addition, analogues of NKB demonstrate strong antitumor activities with no significant side effects in vivo. By activating NK3R in vitro, they exhibit relevant anti-migration effects on Human Umbilical Vein Endothelial Cells (HUVECs) [67]. NKA also regulates pain response, dilation of blood vessels, and vascular permeability by binding to NK2R. Moreover, the poor survival of colorectal cancer patients is correlated with the overexpression of NK2R. It was shown that NK2R-overexpressing murine colorectal carcinoma (CT26) cells demonstrated enhanced tumorigenesis and metastatic colonization in liver and lung, after their inoculation into mice. Consistently, the administration of an NK2R antagonist leads to the suppression of tumorigenesis [68]. Noteworthily, NK2R polymorphism predicts lymph node metastasis in colorectal cancer patients, while VEGFR-3, largely restricted to lymphatic endothelial cells, may play an important role in the regulation of lymphangiogenesis [69,85]. Additionally, NK1R is involved in carcinogenesis. It has been shown that cancers use SP signaling through NK1R to promote the proliferation and survival of cancer cells and to release soluble mediators to promote tumor growth or metastasis. Various tumor types such as breast cancer, colon, glioblastoma, hepatoblastoma, leukemia and pancreatic ductal adenocarcinoma cancer overexpress NK1R [115,116,117,119,120,121,122]. NK1R also promotes non-small cell lung cancer progression through transactivation of EGFR phosphorylation and regulating the intracellular signaling of Extracellular Signal-Regulated Kinase 1/2 (ERK1/2) and Protein Kinase B (Akt). The activation of NK1R stimulates the expression and activities of Matrix Metalloproteinases (MMPs) such as MMP2 and MMP14, involving the migration of glioblastoma, melanoma, and breast cancer cells [71]. Moreover, NK1R antagonists specifically inhibit tumor cell proliferation, angiogenesis, and the migration of tumor cells (including invasion, infiltration and metastasis) [70]. One of the best known NK1R antagonists is aprepitant. This drug is used in the treatment of esophageal squamous cell carcinoma. It induces cancer cells’ growth inhibition by arresting cells in the G2/M phase of cell division, and causes apoptotic cell death. Moreover, it exerts its potent antitumor effects through the suppression of the Phosphoinositide 3-Kinase/Protein Kinase B (PI3K/Akt) axis, and its downstream effector molecules [123]. NK1 receptor antagonists can be also used in the treatment of Central Nervous System (CNS) tumors, which are difficult to treat and are associated with a very bad prognosis because of highly selective blood–brain barrier (BBB) permeability. The BBB is known to restrict common treatments. However, tachykinins, in particular SP, have been implicated in early blood–brain barrier disruption via neurogenic inflammation in a number of other CNS pathologies. The NK1R antagonists are supposed to reduce BBB opening, peritumoral oedema, angiogenesis and tumor growth [124]. Recent studies have showed that a high NK1R expression level is also associated with a high tumor grade and high Ki-67 index (high cell division) in breast cancer. It was concluded that the difference in the expression rate of NK1R results from different isoforms of NK1R–NK1-FL (the full length) and NK1-Tr (truncated, without 96 residues in its cytoplasmic end). Unfortunately, it is still unclear how NK1-FL or NK1-Tr affects tumorigenesis, including the formation and progression stages. However, it is known at this point in time that NK1R-FL expression levels are obviously reduced in breast cancer cell lines and tumor tissues. Contrarily, they are significantly overexpressed in normal breast tissues, while the NK1R-Tr form is highly expressed in breast cancer cells and tissues. Despite the extensive studies on the role of NK1R in cancer, insufficient information is available, especially regarding its role in breast cancer and the molecular mechanisms of its progression. Nevertheless, NK1R seems to be a promising biomarker and therapeutic target in breast cancer [72].

**Figure 4:**
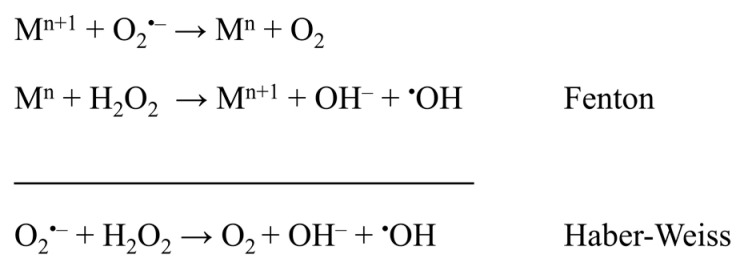
Fenton and Haber–Weiss reactions as a source of oxidative stress, where M = Fe, Cu, and n = charge of metal ion [126,127].

Noticeably higher copper levels in blood plasma (29–56 μM) and increased levels of serum iron (14–33 μM) were also observed in women with PE. Contrarily, lower concentrations of copper (15–26 μM) and iron (7–17 μM) were marked in healthy women [56,57,58]. Notwithstanding, the zinc concentration in blood plasma (11–13 μM) remains similar to that of healthy women; however, the Cu/Zn ratio increases significantly. Moreover, it was suggested that iron may play an important role in the pathophysiology of PE. Most likely, ischaemic placental tissue is a primary source of potentially toxic iron in PE. Released iron species may be involved in the Fenton reaction and contribute to exacerbated lipid peroxidation and endothelial cell injury (Figure 4) [125]. Copper can also undergo a Fenton reaction and, like iron, produce highly reactive hydroxyl radicals. It was also concluded that generated hydroxyl radicals can begin the lipid peroxidation process which may cause endothelial cell damage [57].

Noteworthily, elevated iron levels in pregnant women are considered as a factor qualifying for further observation for preeclampsia. Additionally, copper levels are indicated as markers of preeclampsia severity [53,54]. In the light of recent studies, metal ions are also essential for the growth and proliferation of cancer cells. It was found that metal removal exerts an antiproliferative effect. Therefore, metal ions in biochemical systems became a target for the design of new pharmaceuticals which may perform a key role in potential next generation anticancer drug development strategy. New strategies limiting off-target toxicity, reducing reoccurrence rates, and overcoming or suppressing drug resistance include the use of transition metal chelators, pro-chelators and ionophores to selectively alter the concentrations of iron, copper, and zinc in cancer cells [77]. Worryingly, natural agents could have the opposite effect and increase metal delivery, thus promoting the growth of neoplasm [7].

## 5. Tachykinins Complexes with Trace Elements

**Figure 5:**
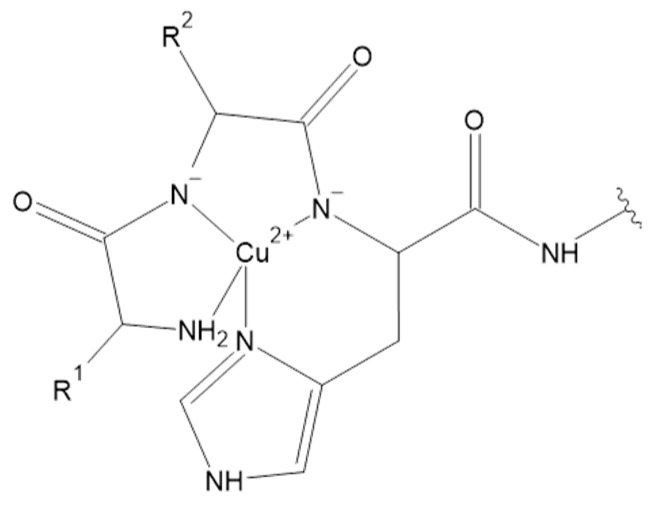
ATCUN motif in Cu(II) complexes [131].

New anti-cancer therapies exploit the ability of transition metal ions to bind various ligands. The resulting complexes have a strong effect on the basic structure and function of cells [128,129]. According to Hard-Soft Acid-Base (HSAB) Theory, Cu(I) is a soft acid, whereas Cu(II), Fe(II) and Zn(II) belong to intermediate acids, while Fe(III) is a member of hard acids. As is known, hard molecules are small, have high charge states and are weakly polarizable. Intermediate and soft molecules are bigger, have lower charge states and are stronger polarizable. Hard acids react faster and form stronger bonds with hard bases, while intermediate and soft acids react faster and form stronger bonds with intermediate and soft bases. Specific metal ions coordination plays a crucial role in the biological function of the metal-bound proteins. Peptides are versatile ligands, as evidenced by the fact that more than 30% of the proteins present in the cell coordinate at least one metal ion [31].The donor groups appropriate for the above-mentioned metal ions constitute various moieties in the natural ligands, including imidazole nitrogen atoms of histidine, side-chain carboxyl groups of aspartate and glutamate, peptide backbone nitrogen and carbonyl groups, as well as the sulfur atoms of cysteine or methionine [130]. There are also particular motifs that interact with metal ions extremely strongly, such as the Amino Terminal Cu(II)– and Ni(II)–Binding Site (ATCUN) motif. This small metal-binding site consists of free N-terminal, histidine residue in the third position of the peptide sequence, and two intervening peptide nitrogens (Figure 5) [131]. As a result of metal ion binding, this complex forms three fused chelate rings (5, 5- and 6-membered ones), which due to the chelating effect are the most stable ones among all peptide ligands [132,133]. It has been postulated that ATCUN motif-bound Cu(II) cleaves DNA or proteins by the production of hydroxyl radicals [134]. Further experiments that insert ATCUN motifs in the N-terminal regions of specific proteins have demonstrated significant DNA cleavage and exhibit antitumor activity [135,136]. Although the ATCUN motif is present in many human peptides and proteins, their role in Cu homeostasis and characteristics of formed complexes have been studied for only a minority of them [137,138,139,140,141]. 

Interestingly, the ATCUN motif is also present in NKB (DMHDFFVGLM–NH_2_) and its complex with Cu(II) ion was widely analyzed both in the water environment and in membrane-mimicking medium [141,142,143]. Contrary to the ATCUN paradigm, some papers suggested unusual complex formation, with metal to ligand stoichiometry as 1:2. In proposed Cu(II)(NKB)_2_ complex species, both peptide molecules are coordinated to copper ions through their N-terminal amine and His imidazole nitrogen atoms [143]. However, the standard 1:1 metal to ligand stoichiometry has recently been proposed based on mass spectrometry experiments [142] and spectroscopic measurements supported with Density Functional Theory (DFT) [141]. In normal pH range of blood, NKB has a high affinity for Cu(II) ions and forms CuH_–2_L complex species with 1:1 metal to ligand stoichiometry, in the presence of micelles and in their absence. It has a {4N} ATCUN coordination type. Furthermore, Cu(II)-NKB in water has a lower dissociation constant, but the interaction with the membrane-mimicking medium weakened the binding nearly one thousand-fold. The stability constants determined for NKB and its N-terminal tetrapeptide allowed the discovery of whether NKB might be able to bind Cu(II) ions in vivo. Considering the average HSA concentration and speciation of Cu(II) ions in blood serum, it was assumed that half of the Cu(II) excess present in PE is shifted to the HSA/NKB system. Results clearly indicate that the total level of Cu(II)-NKB complex in the blood serum may increase more than 100 times during PE. Moreover, recent studies suggest that NKB may affect the copper transport in the central nervous system. The significant loss of the Cu(II) affinity of NKB upon its binding to SDS makes NKB a potentially good Cu(II) delivery agent for the hCtr1 receptor. It seems that the negatively charged phospholipid membrane may act as a switch for biological copper delivery [141]. Moreover, complexes of Cu(II) with another tachykinin, NKA (HKTDSFVGLM–NH_2_), were also investigated. Cu(II)-NKA also forms 1:1 compounds in the physiological pH range of blood. The presence of CuH_2_L was confirmed, but the dimeric Cu_2_H_2_L_2_ species was also found. In the CuH_2_L, coordination takes place through the N-terminal nitrogen atom and imidazole nitrogen of histidine residue in the first position of the peptide chain. However, the formation of the dimeric Cu_2_H_2_L_2_ species is observed through Gly-Gly-like coordination, with the imidazole nitrogen acting as a bridging ligand. Moreover, the susceptibility of NKA to oxidative damage was studied and the products of the copper(II)-catalyzed oxidation of the peptide in the presence of hydrogen peroxide were determined. In general, the metal-catalyzed oxidation reaction targets amino acids in the metal binding pocket of proteins. Noteworthily, the copper(II)-catalyzed oxidation of NKA after 24 h in 37 °C in the presence of hydrogen peroxide (a metal to hydrogen peroxide molar ratio of 1:1) mainly occurs in the methionine and histidine residues. The products of oxidation include, i.a., NKA with oxidized methionine residue (Met-10) to methionine sulfoxide. In other forms, Met-10 was oxidized to methionine sulfoxide and next to sulfone, and His-1 to 2-oxo-His. In addition, under these conditions, peptide bonds cleavage between His-1 and Lys-2, Lys-2 and Met-10, Asp-4 and Ser-5 and decarboxylation of Asp-4 and deamination of His-1 was observed [144]. Furthermore, the Cu(II) complexes with the next tachykinin were studied. However, for Cu(II) and SP (RPKPQQFFGLM–NH_2_), only MALDI Mass Spectrometry (MALDI-MS) measurements were performed. The results suggest the binding of three Cu(II) ions by one SP molecule. Met-11, Leu-10 and Lys-3 were found on the anchoring sites for Cu(II) in SP, according to calculated binding energies determined by DFT. Noteworthily, the pH of the studied solutions was in the range of 2 to 3 [145]. It was also suggested that SP is not able to bind Cu(II) ions selectively in a solution environment at a physiological pH. Most probably, Cu(II) ions are coordinated by water molecules and at least one nitrogen atom, likely the SP N-terminus [143]. However, further studies are needed to determine the coordination sphere of SP, because donor groups are present not only in amino acid side chains, but also in the peptide main chain. The main chain donors include the N-terminal amine, the C-terminal carboxylate and nitrogen atoms of intervening peptide groups [146]. Noteworthily, in the second position of SP sequence L-proline residue is observed. This amino acid acts a ‘break-point’ in the coordination of metal–peptide complexes, and therefore hinders further coordination to the following donor atoms [147].

The low oxidation state metal ion complexes with tachykinins are not well described. Only cuprous complexes with NKB were studied. This neuropeptide binds Cu(I) in the SDS medium. Complex formation occurs after the addition of Cu(I) chloride to the NKB solution. However, the Cu(II) complex may undergo a reduction which results in the formation of Cu(I)-NKB. It is well known that copper functions as a cofactor in enzymes and catalyzes a wide variety of redox reactions due to its ability to cycle between those two oxidation states. The redox property of these metal ions have a potential to cause toxicity if copper homeostasis is not maintained. At pH 6.8, NKB binds Cu(I) ions in a 1:1 stoichiometry. Cu(I) binding involves thioether coordination via Met-2 and Met-10 and an imidazole nitrogen donor atom from His-3. Interestingly, this coordination mode leads to a substantial conformational change of NKB, and some of its helical structure is lost. Because C-terminal Met-10 in NKB binds to the NK3R, the complex formation may affect the activity of the neurokinin receptors [148,149].

**Figure 6:**
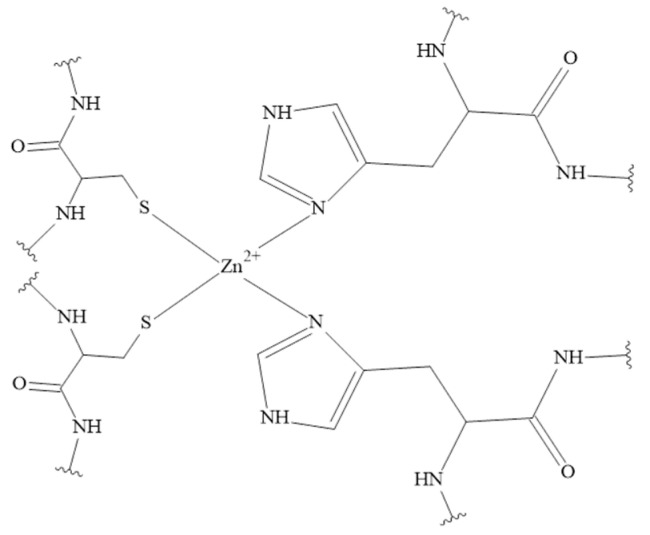
Cys_2_His_2_ type of Zinc Finger Domain in Zn(II) complexes [150].

A commonly known characteristic motif which binds Zn(II) ions, called Zinc Finger Domain (Figure 6) [150], is absent in tachykinin molecules. The motif mainly binds zinc to cysteine residues that are not present in any of the tachykinins discussed so far. However, Zn(II) also interacts with nitrogen and oxygen donor atoms which are present in residues such as histidine or aspartic acid [148,151,152].

Interestingly, there is a known diphenylalanine motif which conserves the specific Zn(II) binding site and prevents hopping of the Zn(II) ion between alternative metal binding sites. The diphenylalanine motif is in the central domain of some neuropeptides, and is present both in NKB and SP. Based on molecular dynamics (MD) results, in the Zn(II) binding site in NKB oxygen atoms deriving from Asp-1 and Asp-4 are involved. The metal ion coordination sphere is fulfilled with two water molecules. Additionally, the Zn(II)-SP complex was characterized by the MD simulations. The coordination mode in SP was based on carboxylic oxygen atoms in the C-terminal with two water molecules. The complex of Zn(II) ions with tachykinin without the diphenylalanine motif, namely NKA, was also determined by MD. The amino acids residues involved in Zn(II) binding were histidine, aspartic acid and methionine. Interestingly, during simulations, Zn(II) ion was selectively transferred among the seven different Zn(II) binding sites [148,153]. To the best of our knowledge, there are no experimental research data concerning this topic. However, results obtained so far support the hypothesis that the diphenylalanine motif plays an important role in maintaining the complex structure and prevents the shifting of the metal ion [148,153]. 

**Figure 7:**
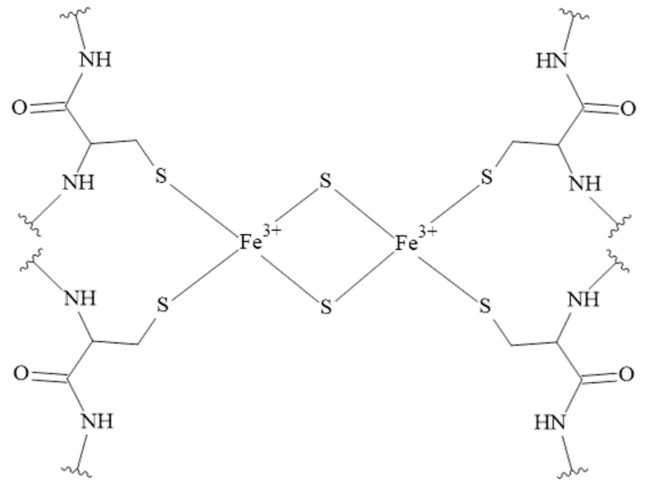
The structure of the rhombic 2Fe-2S cluster. The rhombic-form cluster exhibits two oxidation states: the oxidized status with two Fe^3+^, and the reduced status with one Fe^3+^ and one Fe^2+^ [157].

The iron ions can be bound by various ligands, especially those containing sulfur donor atoms [154,155,156]. Binding to cysteine residues and forming Iron-Sulfur Clusters (ISCs) makes them important redox-active protein cofactors (Figure 7) [157]. Unfortunately, the coordination chemistry of iron ions (on +II and +III oxidation states) with human peptides is a poorly studied area and complexes of Fe(III) ions with tachykinins have not been studied so far.

## 6. Conclusions

**Figure 8:**
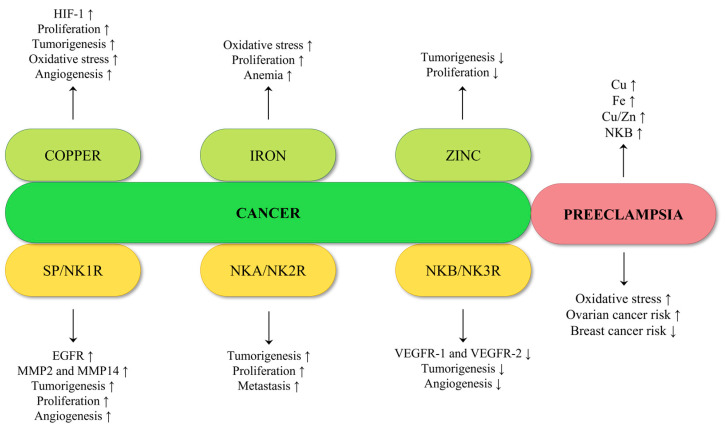
Summary of the effect of copper, iron, zinc, and tachykinins on carcinogenesis, and comparison with features present in preeclampsia.

Pregnancy and the associated changes are normal physiological processes in response to the development of the fetus, whilst cancer progression is a pathological process and extremely difficult to treat. However, proliferation, invasion, and immune privilege are shared between both cancer and pregnancy. Both cancer cells and developing placenta cells create a microenvironment supportive of angiogenesis to establish a nutrient supply. As the pregnancy progresses, metabolic demand increases due to requirements of the growing fetus, and similarly in the case of cancer progression (Figure 8). Trace elements requirements are higher in both cases, e.g., iron ions are necessary for cells’ metabolism and growth. Transition metal ions deficiency can lead to adverse pregnancy outcomes. On the other hand, iron is necessary in the early stages of tumor development, proliferation of neoplastic cells and promotion of metastasis. Noteworthily, copper is necessary for normal iron metabolism and the formation of red blood cells. During pregnancy, it helps to form the fetus’ heart and blood vessels, as well as skeletal and nervous systems. On the other hand, copper allows cancer cells to evade growth suppressors and resist cell death. Therefore, many cancer types exhibit increased intracellular copper levels and altered distribution. However, some metal ions play a protective role during cancer development—for example zinc, which is considered to be critical in the host defense against the initiation and progression of carcinogenesis. Efficient dietary zinc intake is associated with a reduced risk of breast, colorectal, esophageal, gastric and prostatic cancer. It is confirmed that zinc helps to support the immune system and is necessary for many biological functions such as nucleic acid metabolism. Therefore, zinc is essential for normal growth and development and it plays a crucial role during pregnancy.

Tachykinins, such as substance P and neurokinins, belong to the extensively studied neuropeptide family. As neurotransmitters, they carry information between neurons or between neurons and effector cells. Besides their role as neurotransmitters, tachykinins and their receptors are also expressed in several non-neuronal cells. While they are strongly involved in regulating physiological functions, they are also extensively used under pathological conditions including cancer. Noteworthily, many types of tumor cells express neurokinin receptors as well as tachykinins. They influence proliferation, apoptosis, and metastasis of tumor cells in autocrine, paracrine, or neurocrine signaling. The results of multiple studies suggest that tachykinins induce the proliferation and migration of tumor cells and, therefore, the neurokinin receptors’ blockade has antiproliferative and proapoptotic actions. Significantly, overexpression of the short form of NK1R induces the transformation of breast cancer cells. It is suggested that targeting the truncated version of this receptor could be a novel therapeutic strategy.

Many studies pay attention to neurokinin B. This molecule plays key roles in the proper functioning of the female reproductive system, especially during sexual maturation and pregnancy. The physiological level of NKB in the blood increases continuously during pregnancy. Importantly, a much higher level of this peptide was found in pregnant women with PE. This disorder is associated with elevated blood pressure and significant proteinuria. Noteworthily, NKB was proposed to enhance copper delivery to cells. Importantly, PE carries a certain risk of cancer. Recent studies led to the conclusions that women with preeclampsia have higher ovarian cancer risk compared with the woman with normal pregnancy.

From this point of view, it seems to be interesting to investigate the roles of neurokinins and metal ions during this disorder. Therefore, the review highlights the important role of metal ions and their potential complexes with tachykinins in PE and all stages of cancer development. It also combines recent studies on PE and tachykinin related tumorigenesis, and provides information on transition metal ion-tachykinin complexes which could be crucial in these disorders.

## Data Availability

Not applicable.
